# Acetic acid‐induced colitis modulating potential of total crude alkaloidal extract of *Picralima nitida* seeds in rats

**DOI:** 10.1002/iid3.855

**Published:** 2023-05-08

**Authors:** Sarah A. Otu‐Boakye, Kofi O. Yeboah, Eric Boakye‐Gyasi, James Oppong‐Kyekyeku, Prince D. Okyere, Newman Osafo

**Affiliations:** ^1^ Department of Pharmacology, Faculty of Pharmacy and Pharmaceutical Sciences, College of Health Sciences Kwame Nkrumah University of Science and Technology (KNUST) Kumasi Ghana; ^2^ Department of Pharmaceutical Chemistry, Faculty of Pharmacy and Pharmaceutical Sciences, College of Health Sciences Kwame Nkrumah University of Science and Technology (KNUST) Kumasi Ghana

**Keywords:** acetic acid, chronic inflammation, cytokines, inflammatory bowel disease, *Picralima nitida*, ulcerative colitis

## Abstract

**Purpose:**

The total crude alkaloidal extract of *Picralima nitida* seeds (PNE) is known to possess anti‐inflammatory activity among other therapeutic benefits although its benefits in colitis has not been investigated. The current study therefore seeks to investigate the anti‐colitis potential of PNE using acetic acid‐induced colitis model in rats.

**Methods:**

Sprague Dawley rats were treated with oral 500 mg/kg sulphasalazine or 30, 100, and 300 mg/kg of PNE daily for 8 days with induction of colitis on the fourth day with acetic acid. Rats were killed 24 h after the last treatment and whole blood was obtained from the jugular vein for hematological analysis and biochemical assays. Colons were extirpated for assessment of macroscopic and histological damage to the colon.

**Results:**

Treatment with PNE protected against colonic injury induced with acetic acid by decreasing mucosal ulceration, epithelial erosion, inflammatory cell infiltration, and colonic edema. Thus, PNE preserved mucosal architecture and suppressed goblet cells depletion. Moreover, treatment with PNE was associated with improved hematological parameters and reductions in the expression of serum tumor necrosis factor‐alpha, interleukin‐1β, and p38 mitogen‐activated protein kinase. Also, PNE treatment exerted antioxidant effects by reducing nitric oxide production and increasing glutathione levels. In addition, PNE inhibited colonic lipid peroxidation by decreasing myeloperoxidase activity and malondialdehyde production.

**Conclusion:**

It can be concluded that PNE attenuates intestinal oxidative and inflammatory damages following intrarectal acetic acid challenge. Thus, demonstrates potential for use in chronic intestinal inflammatory diseases such as ulcerative colitis.

## Graphical abstract

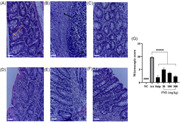

*Picralima nitida* seeds (PNE) suppresses colon inflammation in acetic acid‐induced colitis model in rats. PNE inhibits expression of pro‐inflammatory cytokines. PNE exhibits antioxidant action in its anti‐inflammatory potential.

## 1 INTRODUCTION

Ulcerative colitis (UC) is a complex chronic inflammatory disorder of the large bowel that is associated with a higher tendency of exhibiting a relapsing‐remitting pattern.^1^ Albeit its etiology is not fully understood, it is widely believed that UC occurs as a result of destabilization of intestinal homeostasis and optimal immunosurveillance.^2^ This leads to loss of immune tolerance towards the luminal antigens and the resident microbiota. Thus, an exaggerated and uncontrolled immune response that induces nonspecific mucosal and submucosal chronic inflammation in the rectum, sometimes extending proximally within the colon, ensues.^1^ In addition, inflammatory cytokines secreted by various immune cells stimulate the proliferation of antigen‐specific effector cells which mediate this aberrant immune response principally through activation of the adaptive immune system.^3^ Together with Crohn's disease, these inflammatory bowel diseases (IBDs) are associated with significant global disease burden with increased incidence rates due to global industrialization, urbanization, and improved diagnosis.^4^

In spite of improved diagnosis, UC continues to place significant burden on global healthcare. This is typified by the immensity of treatment challenges, being surgery or pharmacotherapy. Sadly, mortality data in patients with UC reveals about 10% increase in intermediate and long‐term mortality with an even graver outcome in patients diagnosed in childhood or adolescence.^5^ Thus, indicating the failures associated with current clinical management of UC.

For centuries, medicinal plants have proven to be important sources of lead compounds in the treatment of various diseases. Importantly, numerous studies have demonstrated the beneficial and protective effects of natural products in experimental models of colitis.^1^, ^6^ As such, there is a recent heightened interest in natural products from plants, animals, and microorganisms including their extracts and secondary metabolites. One such natural source known for its important role in traditional medicine is the plant *Picralima nitida* (Stapf.) T. Durand & H. Durand of the Family Apocynaceae.

*Picralima nitida* is a low‐growing understory tree commonly known as pile plant and akuamma in Ghana. The plant can grow up to 35 m in height, with a dense crown, and a trunk that reaches a diameter of 5–60 m. The flowers are bisexual and bears ovoid fruits that are yellowish and contain multiple seeds. The seed on the other hand is flat, ovoid to oblong, with smooth surface.^7^ Traditionally, the crushed seeds are administered orally in the treatment of gastrointestinal disorders, skin abscesses and chest infections.^8^ Interestingly, previous studies on the total alkaloidal extract and some specific alkaloids such as pseudoakuammigine has demonstrated significant activity in both acute and chronic inflammatory models^9^ but there is no reported benefit of the extract from the seeds in colitis. Thus, this study aimed to investigate the effect of the total alkaloidal extract of *P. nitida* on acetic acid‐induced UC.

## 2 MATERIALS AND METHODS

### 2.1 Materials

#### 2.1.1 Plant collection and identification

The ripe fruits *P. nitida* were obtained from the botanical gardens of Kwame Nkrumah University of Science and Technology (KNUST) (6°41′7" N 1°33′48" W), Kumasi in January 2021. The plant was identified and authenticated at the Department of Herbal Medicine, KNUST.

#### 2.1.2 Extraction of the plant material

The total crude alkaloidal fraction was extracted as described by Woode et al.^9^ Briefly, the dried seeds of *P. nitida* were milled to a fine powder and 1.8 kg of the powder soaked in 10 L of petroleum ether for 48 h to eliminate fats, oils, and waxes present. The extract was filtered and concentrated using a rotary evaporator (R‐210; BUCHI) at 50°C. The process was repeated three times to further eliminate fats, oils and waxes. The marc obtained after filtration was dried on a tray at room temperature and subsequently dissolved in 7 L of 6%^v^/_v_ acetic acid and left overnight to liberate the total crude alkaloids from the defatted seed. The extract was filtered and the filtrate was basified with concentrated ammonia to a pH of 9 to precipitate the alkaloids. The basified extract was then transferred into a separating funnel and chloroform was added in a of ratio 2:1 (two parts of the basified extract to one part of chloroform) to dissolve the precipitated alkaloids. Subsequently, the chloroform fraction was separated using a separating funnel and was concentrated at 50°C using a rotary evaporator. The remaining solvent was evaporated using a water bath to obtain the total crude alkaloidal fraction of *P. nitida* seeds with a yield of 4.83% ^w^/_w_.

$$\%\;\mathrm{Yield}=\frac{A_{1}}{A_{0}}x\;100\%,$$

where *A*_0_ was the mass of the seed sample and *A*_1_ was the mass of the total crude alkaloidal extract.

#### 2.1.3 Animals

Male Sprague Dawley rats (150–200 g) were procured and housed in stainless steel cages (34 × 47 × 18 cm) with soft wood shavings as bedding, at the animal house of the Department of Pharmacology, Kwame Nkrumah University of Science and Technology, Kumasi, Ghana. Rats were given adequate time to acclimatize and were maintained on a standard chow diet with access to clean water ad libitum. Animals were handled appropriately throughout the study, as recommended by the Animal Welfare Regulations (USDA 1985; US Code, 42 USC 289d) and the Public Health Service Policy on Humane Care and Use of Laboratory Animals (PHS 2002). Ethical clearance for this study was obtained from the Animal Research Ethics Committee (AREC) of KNUST for the work using rodents (reference: KNUST 0014).

#### 2.1.4 Drugs and chemicals

Sulphasalazine was bought from Pfizer Limited; *o*‐dianisidine dihydrochloride, acetic acid, thiobarbituric acid, and trichloroacetic acid were from Sigma‐Aldrich Chemical Company; halothane was purchased from Piramal Enterprises Limited; hydrogen peroxide was from Bell's Healthcare; hematoxylin and eosin (H and E) stain was purchased from Abcam; concentrated ammonia, petroleum ether, and chloroform were obtained from VWR International Leuven (Belgium); rat tumor necrosis factor‐alpha (TNF‐α), interleukin‐1β (IL‐1β), glutathione, nitric oxide, and p38 mitogen‐activated protein kinase (p38 MAPK) enzyme‐linked immunosorbent assay (ELISA) kits were purchased from Shanghai Enzyme‐linked Biotech Limited (China).

### 2.2 Methods

#### 2.2.1 RP‐HPLC method development

A gradient chromatographic mode was employed to obtain the RP‐HPLC fingerprint of the extract of *P. nitida*. The analysis was performed on a Luna 3 μ C18(2) (150 × 4.60 mm) column from Phenomenex using mobile phase of 0.05% TFA (A) and methanol (B) at a flow rate of 1.0 mL/min with the column kept at an ambient temperature. The gradient elution was achieved by equilibration of the column for 1 min with 95% (A) and 5% (B) preceding injection of the extract. This was followed by a further run with the same composition for 3 min. A composition of 20% (A) and 80% (B) was run for 25 min and the last 6 min with 95% (A) and 5% (B). The detection of the eluate by photodiode array (PDA) was monitored at 225 nm. The chromatogram recorded showed well‐resolved peaks. The procedure was repeated three consecutive times and the results were found to be reproducible.

#### 2.2.2 Acetic acid‐induced UC in Sprague Dawley rats

Colitis was induced with acetic acid as described by Osafo et al.^10^ and Tagne et al.^11^ Briefly, male Sprague Dawley rats (150–200 g) were randomly grouped into five (*n* = 7). Treatment was done to various groups as follows:

Group I: normal saline (0.9% ^w^/_v_) *p.o* for 8 days.

Group II: normal saline (0.9% ^w^/_v_) *p.o* for 8 days, and 1 mL 4.0% ^v^/_v_ acetic acid intrarectally on day 4.

Group III: sulphasalazine (500 mg/kg *p.o*.) for 8 days and 1 mL 4.0% ^v^/_v_ acetic acid intrarectally on day 4.

Groups IV–VI: *P. nitida* total crude alkaloidal extract (PNE) 30, 100, and 300 mg/kg *p.o*. respectively^9^ for 8 days and 1 mL 4.0% ^v^/_v_ acetic acid intrarectally on Day 4.

Over the period of 8 days, changes in body weight were recorded. The rats were euthanized with an overdose of halothane 24 h after the last doses of various treatments were administered. Colons of rats were extirpated under aseptic conditions and luminal contents were washed off with cold phosphate‐buffered saline.

#### 2.2.3 Disease activity index (DAI)

Following induction of colitis, a modified DAI scoring method was used to assess the consistency and condition of rats' feces.^12^ The DAI scores (0–4) were computed based on the consistency and the presence of blood in feces as described by Nagib et al.^12^ (Table 1).

**Table 1:** Disease activity index.

Score	Stool Consistency	Blood in feces
0	Normal	Negative
1		Occult blood ±
2	Loose	Occult blood +
3		Occult blood ++
4	Diarrhea	Gross blood

*Note*: Blood in feces (0, negative; 1: ±, 2: +, 3: ++, 4: gross).

#### 2.2.4 Colon edema determination

Colon injury was induced in rats with acetic acid as described in Section 2.2.2. At the end of the 8‐day period, colons were ectomized and cut open by longitudinal incisions. The excised colons were then washed thoroughly with normal saline, and the weight and length of colon samples were measured. Colon weight‐to‐length ratio, a measure of intestinal edema, was computed as described by Yeboah et al.^6^

#### 2.2.5 Macroscopic assessment of colon

Acetic acid‐induced colon injury was induced in rats as described in Section 2.2.2. At the end of Day 8, the rats were euthanized by overdose of halothane and the large bowels were excised. Macroscopic damage to the colon was assessed by two observers blinded to the study as described by Camuesco et al.^13^ Briefly, colon damage was assessed using a 10‐point scale based on the degree of inflammation and ulceration as follows: 0, no damage; 1, hyperemia with no ulcers; 2, linear ulceration without significant inflammation; 3, linear ulceration with inflammation at a single site; 4, ≥2 sites of inflammation or ulceration; 5, ≥2 major sites of ulceration and inflammation at a single site of inflammation or ulceration covering greater than 1 cm along the length of the colon; 6–10, if damage extends >2 cm along the length of the colon, score is increased by 1 for any additional centimeter involved.^14^

#### 2.2.6 Histopathological assessment of colon

To evaluate microscopic colon damage by light microscopy, distal colon samples were fixed in 10% formaldehyde, embedded in paraffin, cut into transverse sections and mounted on glass slides. Subsequently, the sections were stained with hematoxylin and eosin stain and six random fields of view were observed to evaluate microscopic damage using a cumulative semiquantitative scoring scale of 0–11 as described by Appleyard and Wallace.^15^ Briefly, thickening of muscularis mucosae (0–3), loss of mucosal architecture (0–3), inflammatory cell infiltration (0–3), goblet cell depletion (0–1), and crypt abscess formation (0–1) were assessed for each sample.

#### 2.2.7 Hematological assessment

Whole blood was taken from the jugular vein at the end of Day 8. Full blood count was carried out using a hematology analyzer (YSTE880‐Guangzhou Yueshen Medical Equipment Co. Ltd.).

#### 2.2.8 Assay of serum levels of TNF‐α, IL‐1β, GSH, NO, and p38 MAPK

Colon injury was induced in rats with acetic acid as described in Section 2.2.2. At the end of the 8‐day period, whole blood was taken from the jugular vein and centrifuged at 600 × *g* for 30 min at 4°C to obtain serum. Following the manufacturer's protocol, rat TNF‐α, IL‐1β, GSH, NO, and p38 MAPK ELISA kits were used to assay the serum concentrations of TNF‐α, IL‐1β, GSH, NO, and p38 MAPK, respectively.

#### 2.2.9 Myeloperoxidase and malondialdehyde assay

To obtain a 10% homogenate, 1 g of colon samples from the various treatment groups in both models were homogenized with a Potter‐Elvehjem homogenizer (Ultra‐Turrax T25, Janke & Kunkel IKA‐ Labortechnik) in cooled 10 mL of 0.01 M Tris‐HCl solution with pH 7.4. Myeloperoxidase (MPO) and malondialdehyde (MDA) assays were performed on the homogenate.

##### Myeloperoxidase assay

MPO activity was measured using a modified *o*‐dianisidine method. Briefly, 1 g of colon samples were homogenized in 10 mL of cooled 0.01 M Tris‐HCl solution (pH 7.4) with Potter‐Elvehjem homogenizer (Labortechnik). The supernatant of the resulting homogenate was added to the assay mixture containing 0.1 M phosphate buffer (pH 6), 0.167 mg/mL *o*‐dianisidine hydrochloride and 0.0005% hydrogen peroxide to a volume of 3.0 mL. Changes in absorbance at every minute over a total period of 10 min were measured at a wavelength of 460 nm. Each measurement was done in duplicate. A unit of MPO was estimated as that producing absorbance increment of 0.001 min**^−^** ^1^ and specific activity measured as mU/mg protein.

##### Malondialdehyde assay

Homogenates of colon samples were obtained as described above and used to assay MDA levels as described by Heath and Parker.^16^ Briefly, 1 mL aliquot of supernatant was added to a mixture of 20% trichloroacetic acid and 0.5% thiobarbituric acid to make a final volume of 3 mL. The mixture was heated at 95°C for 30 min and immediately cooled on ice bath after which the mixture was centrifuged at 10,000 × *g* for 10 min. The supernatant was removed into a fresh tube and absorbance was measured at 532 nm. The nonspecific absorption value at 600 nm was deducted from the absorbance read at 532 nm. The concentration of MDA was estimated with extinction coefficient 155 mM^‐1^cm^‐1^.

### 2.3 Statistical analysis

Data were presented as mean ± SEM. Parametric data were subjected to analysis of variance (ANOVA). Time‐course curves were analyzed by two‐way ANOVA with Tukey's multiple comparisons test. All other experimental data were analyzed using one‐way ANOVA followed by Tukey's multiple comparisons test. Graphs were plotted using GraphPad Prism for Windows version 8.01 (GraphPad).

## 3 RESULTS

### 3.1 RP‐HPLC

**Figure 1:**
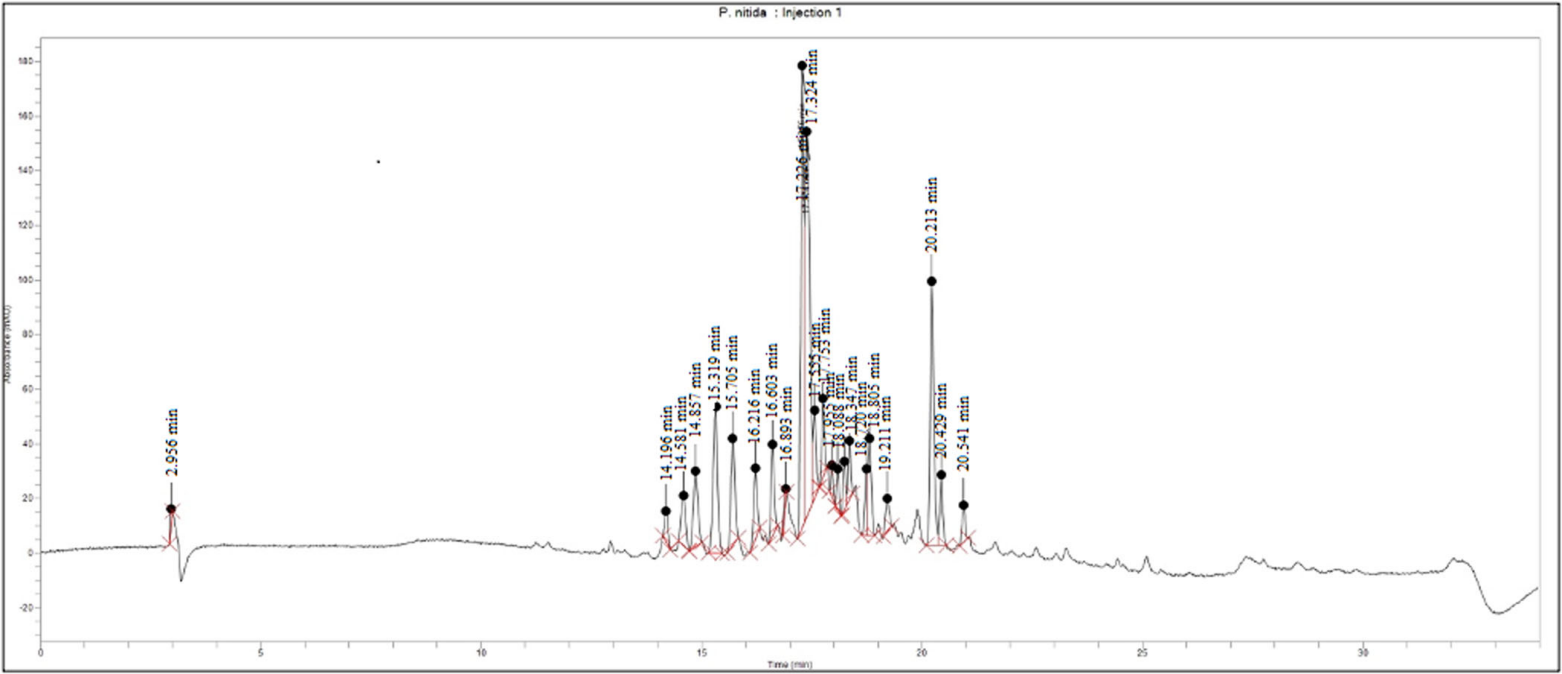
Chromatographic fingerprint of *Picralima nitida* seeds.

The chromatogram obtained showed well‐separated peaks with the most prominent peak at a retention time of 15.32 min. Most of the peaks recorded retention times between 14 min and 16 min. The secondary metabolites in the extract may therefore be chemically related compounds (Figure 1).

### 3.2 Disease activity index (DAI), changes in body weight, and colon weight‐to‐length ratio determination

**Figure 2:**
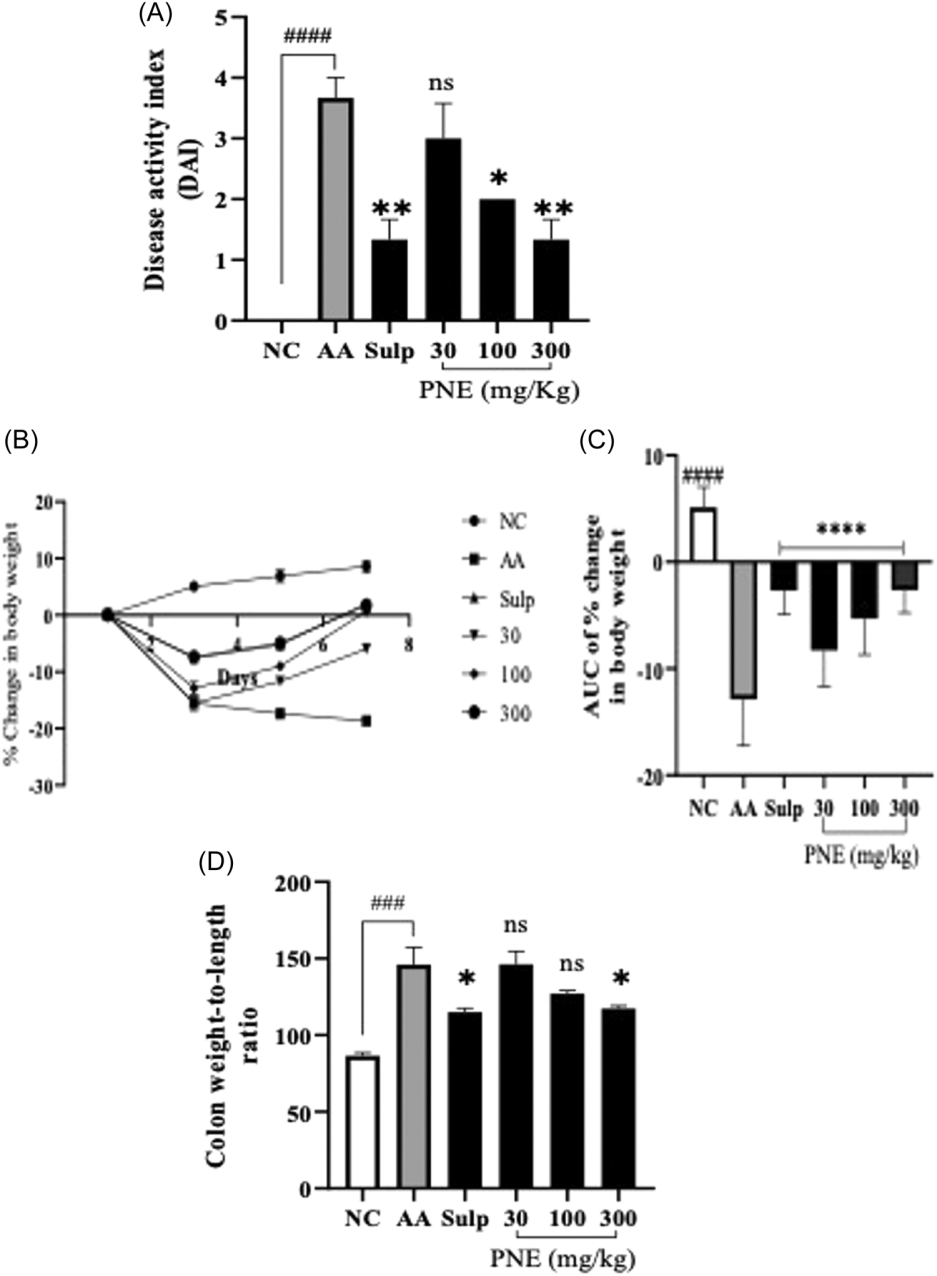
Effect of *Picralima nitida* seeds (PNE) on disease activity index (0–4). (A) Changes in rat body weight (B, C) and colon weight/length ratio (D) in acetic acid‐induced rat inflammatory bowel diseases (IBDs). ^####^ *p* < .0001 in comparison to naïve control (NC). ^####^ *p* < .0001, ^###^ *p* = .0002 in comparison to NC. *p < .05 and ^ns^ *p* > .05 (on A), ****p < .0001, *p = .0372, **p < .01 and ^ns^ *p* > .05 (on D) in comparison to acetic acid control (AA) (one‐way analysis of variance and Tukey's multiple comparisons test, *n* = 7). Sulp, Sulphasalazine.

Intrarectal administration of acetic acid in the colitis control resulted in colonic injury characterized by loose bloody stools and associated a significantly (*p* < .0001) higher DAI score of 3.33 ± 0.33 compared to zero disease activity in the noncolitis control rats (Figure 2A). Thus, confirming the successful induction of colitis in the colitis groups. Treatment with sulphasalazine significantly (*p* < .05) suppressed disease activity with DAI score of 1.0 ± 0.01 compared to 3.33 ± 0.33 DAI of the colitis control group (Figure 2A). Likewise, treatment with PNE at doses 100 and 300 mg/kg significantly (*p* < .05) reduced DAI scores to 2.0 ± 0.57 and 1.33 ± 0.33, respectively. Administration of 30 mg/kg of the extract, however, resulted in nonsignificant (*p* > .05) reduction in DAI scores compared to the colitis control group (Figure 2A). Treatment with PNE at doses of 100 and 300 mg/kg is thus effective in significantly reducing DAI scores following induction of colon injury with acetic acid.

Intrarenal administration of acetic acid resulted in significant (*p* < .0001) body weight loss in colitis control compared to noncolitis control (Figure 2B,C). Administration of sulphasalazine in the positive control significantly (*p* < .0001) reduced drastic loss of body weight compared to the colitis control (Figure 2B,C). PNE‐treated rats demonstrated a steady rise in body weight after induction of colon injury (Figure 2B) with significant (*p* < .0001) reduction in loss of body weight over the entire period of study compared to the colitis control rats (Figure 2C).

Induction of colitis in the colitis control rats resulted in a significant (*p* < .0001) increase in the mean colon weight‐to‐length ratio (146.0 ± 11.31 g/cm) compared to 86.53 ± 2.02 g/cm in noncolitis control group (Figure 2D). Administration of sulphasalazine to the positive control group resulted in significant (*p* < .05) reduction in colon edema with mean colon weight‐to‐length ratio of 123.1 ± 1.59 g/cm compared to 146.0 ± 11.31 g/cm of the colitis control (Figure 2D). Test group that received PNE at dose of 30 and 100 mg/kg exhibited no significant (*p* > .05) reductions in mean colon weight‐to‐length ratio when compared to the colitis control group. Conversely, treatment with 300 mg/kg of the extract significantly reduced colon edema with mean colon weight‐to‐length ratio of 117.3 ± 1.91 g/cm compared to colitis control group (Figure 2D).

### 3.3 Macroscopic assessment of colon

**Figure 3:**
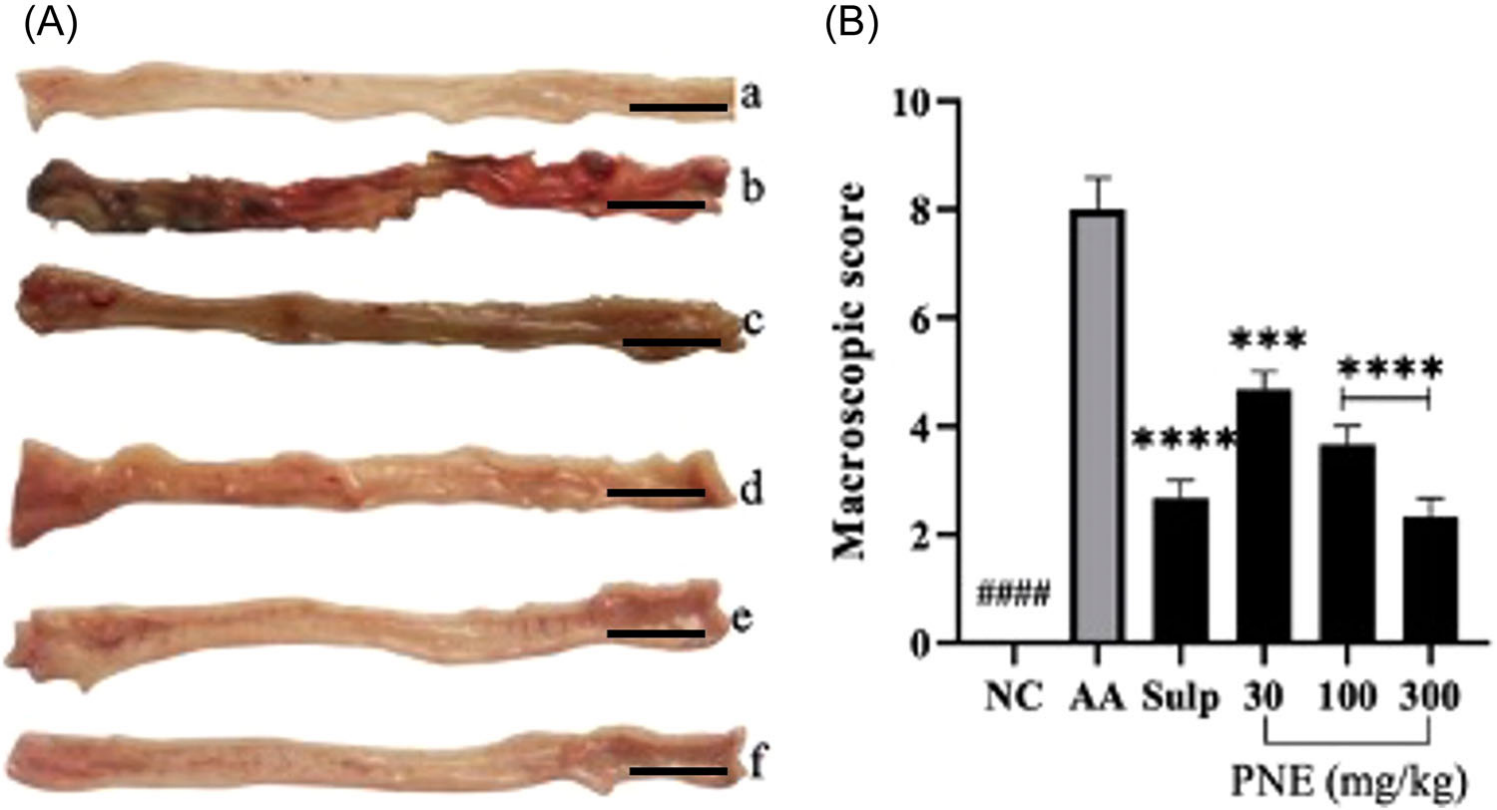
Effect of *Picralima nitida* seeds (PNE) on macroscopic damage in acetic acid‐induced inflammatory bowel diseases (IBDs). (A) Representative slides of colon (a, naïve control; b, AA control; c, AA + 500 mg/kg sulphasalazine; d, AA + 30 mg/kg PNE; e, AA + 100 mg/kg PNE; f, AA + 300 mg/kg PNE). (B) Macroscopic scores (0–10) for colon samples. ^####^ *p* < .0001 when compared with naïve control (NC). ***p = .0003 and ****p < .0001 in comparison to acetic acid control (AA) (one‐way analysis of variance and Tukey's multiple comparisons test, *n* = 7). Micron bar represents 75 mm.

Colitis control rats exhibited severe inflammation and ulceration of the colon. This is evident by the significant elevation in mean macroscopic score (8.0 ± 0.57) compared to zero macroscopic damage in the noncolitis control group (Figure 3). Macroscopic damage was reduced following treatment with sulphasalazine in the positive control group with mean macroscopic score of 2.67 ± 0.33. This represented a significant (*p* < .05) reduction in colon inflammation and ulceration, compared to a mean score of 8.0 ± 0.57 in the colitis control group. Similarly, treatment with PNE reduced mucosal erythema, erosions, and ulceration observed following treatment with PNE (Figure 3A). PNE at all doses (30, 100, 300 mg/kg) significantly (*p* < .001) suppressed macroscopic damage to the colon with mean scores 4.67 ± 0.33, 3.7 ± 0.31, and 2.33 ± 0.33 compared to colitis control (Figure 3B).

### 3.4 Histopathological assessment and microscopic scores of colons

**Figure 4:**
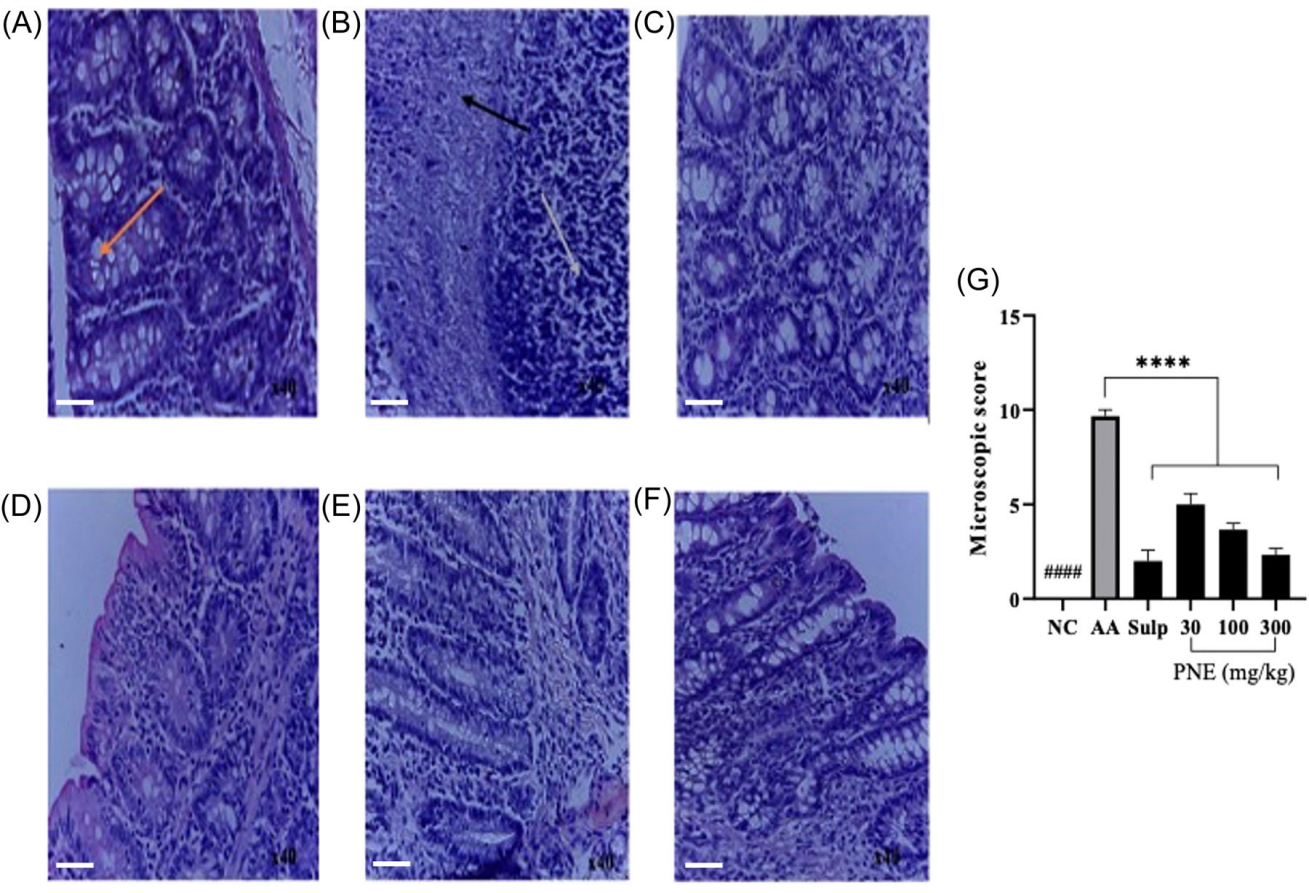
Histopathology and microscopic scores of acetic acid‐induced IBD in rats. Histological sections of colon samples. (A) Naive control; (B) acetic acid control; (C) sulphasalazine 500 mg/kg; (D) *Picralima nitida* seeds (PNE) 30 mg/kg; (E) PNE 100 mg/kg; (F) PNE 300 mg/kg (magnification ×40). Region showing goblet cells (orange arrow), cellular infiltration (gray arrow), muscle thickening (black arrow). ^####^ *p* < .0001 in comparison to naïve control (NC). ****p < .0001 in comparison to acetic acid control (AA) (one‐way analysis of variance and Tukey's multiple comparisons test, *n* = 7). Sulp, Sulphasalazine. Staining was made with haematoxylin and eosin; Micron bar represents 100 μm.

Induction of colitis with acetic acid in the colitis control resulted in profound colon inflammation characterized by goblet cell depletion, formation of crypt abscesses, massive thickening of the submucosa, ulceration, and lymphocyte infiltration all of which were scarce in the noncolitis group (Figure 4A,B). Inflammatory cell infiltration, mucosal thickening, and goblet cell depletion were all reduced following treatment with sulphasalazine (Figure 4C). Likewise, treatment with PNE at all doses decreased gross mucosal damage, reduced epithelial erosion, and decreased inflammatory cell infiltration, mucosal thickening, and goblet cell depletion (Figure 4D–F). Quantitatively, intrarenal administration of acetic acid in colitis control significantly (*p* < .0001) increased mean microscopic scores of 9.33 ± 0.35 compared to noncolitis control group (Figure 4G). Treatment with sulphasalazine significantly (*p* < .0001) reduced microscopic damage with mean microscopic score of 2.00 ± 0.57 compared to the colitis control. PNE at all doses significantly (*p* < .0001) decreased mean microscopic scores to 5.0 ± 0.57, 3.67 ± 0.33, and 2.33 ± 0.33, respectively (Figure 4G).

### 3.5 Hematological assessment

Full blood count obtained following analysis of whole blood samples collected from the jugular vein of the animals showed significant imbalances in hematological parameters in colitis control and noncolitis control groups. Induction of colon injury resulted in significant (*p* < .0001) elevation in plasma white blood cell (WBC) and lymphocyte (LYM) concentrations, with a significant (*p* < .0001) reduction in hemoglobin (HGB) concentrations (Table 2). Conversely, treatment with sulphasalazine significantly (*p* < .0001) reduced WBC and LYM levels, but increased HGB concentration when compared to the colitis control. Similarly, treatment with PNE at all doses resulted in an increase in HGB and platelet and a significant (*p* < .0001) decrease in WBC, LYM, and neutrophil count when compared with the colitis control.

**Table 2:** Effect of *Picralima nitida* seeds (PNE) on hematological parameters in acetic acid‐induced inflammatory bowel diseases (IBDs) in rats.

	Naïve control	Acetic acid control	Sulphasalazine	PNE (mg/kg)		
Parameter			(500 mg/kg)	30	100	300
White blood cell × 10^3^ (cells/μL)	7.9 ± 0.01	13.9 ± 0.02####	9.7 ± 0.01****	10.8 ± 0.01****	6.9 ± 0.01****	5.8 ± 0.01****
Lymphocyte × 10^3^ (cells/μL)	2.2 ± 0.03	5.7 ± 0.01####	2.2 ± 0.02****	4.2 ± 0.02****	2.5 ± 0.01****	0.7 ± 0.01****
NEUT × 10^3^ (cells/μL)	4.3 ± 0.03	5.7 ± 0.01	5.4 ± 0.01	5.5 ± 0.02	2.8 ± 0.05****	3.6 ± 0.08****
RBC × 10^6^ (cells/μL)	5.4 ± 0.11	6.7 ± 0.01####	6.6 ± 0.01	7.1 ± 0.11****	6.3 ± 0.01	7.5 ± 0.01****
PLT × 10^3^ (cells/μL)	727.3 ± 9.84	361.7 ± 3.48####	1047 ± 0.89****	607.0 ± 1.16****	936.0 ± 5.69****	978.7 ± 0.88****
HGB (g/dL)	13.3 ± 0.09	11.7 ± 0.12####	13.6 ± 0.06****	13.9 ± 0.09****	14.4 ± 0.06****	15.1 ± 0.09****
HCT (%)	33.2 ± 0.09	31.3 ± 0.12	32.7 ± 0.09	33.7 ± 0.12	33.8 ± 0.09	39.3 ± 0.15

*Note*: Data are presented as mean ± SEM (*n* = 7).

Abbreviations: HCT, hematocrit; HGB, hemoglobin; NEUT, neutrophil; PLT, platelet; RBC, red blood cell.

^####^ *p* < .0001 in comparison to naïve control (NC);

**** *p* < .0001 in comparison to acetic acid control (one‐way analysis of variance and Tukey's multiple comparisons test).

### 3.6 Serum levels of TNF‐α, IL‐1β, p38 MAPK, NO, and GSH

**Figure 5:**
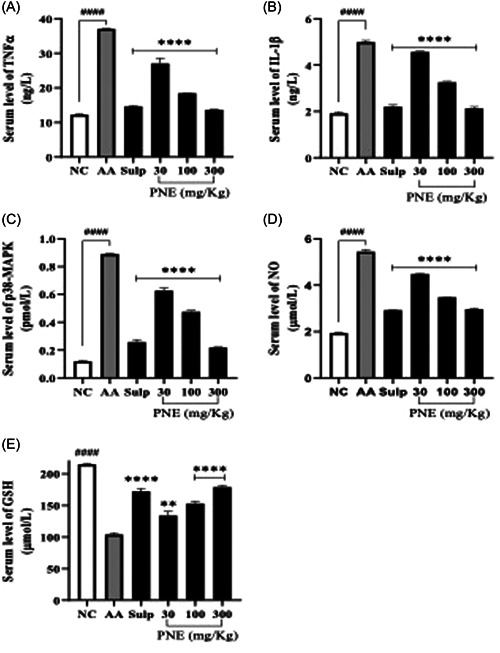
Effect of *Picralima nitida* seeds (PNE) on the production of inflammatory mediators and the occurrence of oxidative damages in acetic‐induced inflammatory bowel diseases (IBDs). Serum levels of tumor necrosis factor‐alpha (TNF‐α) (A), interleukin‐1β (IL‐1β) (B), p38 mitogen‐activated protein kinase (p38 MAPK) (C), nitric oxide (NO) (D), and glutathione (GSH) (E). ^####^ *p* < .0001 in comparison to naïve control (NC). ****p < .001 in comparison to acetic acid control (AA) (one‐way analysis of variance and Tukey's multiple comparisons test, *n* = 7). Sulp, Sulphasalazine.

Colonic injury induced with acetic acid in the disease control group resulted in significant (*p* < .0001) elevation in serum TNF‐α levels with serum concentration of 37.4 ± 0.12 ng/L compared to 12.2 ± 0.21 ng/L in the noncolitis control (Figure 5A). Treatment with sulphasalazine significantly (*p* < .0001) suppressed serum levels of TNF‐α 14.6 ± 0.23 ng/L when compared to the untreated control group. Treatment with PNE at doses of 30, 100, and 300 mg/kg likewise suppressed the rise in serum TNF‐α levels with serum concentrations of 27.1 ± 1.51, 18.4 ± 0.12, and 13.6 ± 0.12 ng/L, respectively. Thus, PNE significantly reduced serum TNF‐α compared to the colitis control (Figure 5A).

Colon acetic acid challenge in the colitis control significantly (*p* < .0001) elevated serum IL‐1β levels (4.9 ± 0.07 ng/L) compared to 1.9 ± 0.07 ng/L of the noncolitis group. in the TNBS and acetic acid control groups to 7.2 ± 0.09 ng/L and 4.9 ± 0.07 ng/L, respectively (Figure 5B). In contrast, treatment with sulphasalazine or PNE at all doses significantly (*p* < .0001) reduced serum concentrations when compared to colitis control. Administration of sulphasalazine or PNE at 30, 100, and 300 mg/kg reduced serum IL‐1β concentrations to 4.6 ± 0.03, 3.3 ± 0.04, and 2.1 ± 0.07 ng/L, respectively.

As shown in Figure 5C, induction of colitis in the colitis control resulted in a significant (*p* < .0001) increase in serum levels of p38 MAPK, 0.9 ± 0.01 pmol/L compared to noncolitis control. Treatment with sulphasalazine significantly (*p* < .0001) decreased serum expression of p38 MAPK to 0.3 ± 0.02 pmol/L compared to 0.9 ± 0.01 pmol/L of the colitis control. PNE treatment at 30, 100, and 300 mg/kg reduced serum expression of p38 MAPK into 0.6 ± 0.02, 0.5 ± 0.01, and 0.2 ± 0.01 pmol/L, respectively. These represent significant (*p* < .0001) reductions in serum p38 MAPK concentrations compared to the colitis control group (Figure 5C).

The serum level of NO in the colitis control was significantly (*p* < .0001) increased compared to the noncolitis control (Figure 5D). Administration of sulphasalazine over the 8‐day period significantly decreased serum levels of NO with mean serum concentration of 2.9 ± 0.11 µmol/L compared to 5.4 ± 0.09 µmol/L of the colitis control. Likewise, treatment with PNE at 30, 100, and 300 mg/kg reduced serum levels of NO into 4.5 ± 0.04, 3.5 ± 0.02, and 2.9 ± 0.03 µmol/L, respectively (Figure 5D).

As shown in Figure 5E, colon inflammation induced with acetic acid significantly (*p* < .0001) reduces serum GSH concentration compared to uninflamed colon with mean serum levels of 104.0 ± 2.85 and 214.9 ± 1.21 µmol/L, respectively. Sulphasalazine significantly (*p* < .0001) elevated serum GSH levels (163.6 ± 4.38 µmol/L) compared to the colitis control. Treatment with PNE at 30, 100, and 300 mg/kg significantly (*p* < .001) improved serum GSH levels with mean serum concentrations of 133.9 ± 6.96, 153.0 ± 3.56, and 178.8 ± 2.84 µmol/L, compared to 104.0 ± 2.85 µmol/L of the colitis control group (Figure 5E).

### 3.7 Myeloperoxidase and malondialdehyde assays

**Figure 6:**
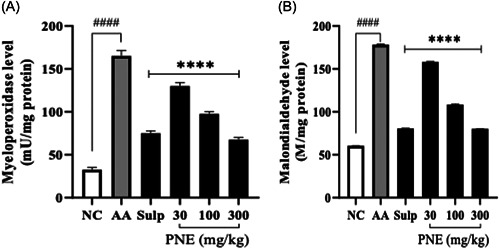
Effect of *Picralima nitida* seeds (PNE) on myeloperoxidase expression and malondialdehyde levels in acetic acid‐induced inflammatory bowel diseases (IBDs) in rats. ^####^ *p* < .0001 in comparison to naïve control (NC). ****p < .0001 in comparison to acetic acid control (AA) (one‐way analysis of variance and Tukey's multiple comparisons test, *n* = 7). Sulp, Sulphasalazine.

The activity of MPO was detectable in the noncolitis control (32.50 ± 2.5 mU/mg protein) but was significantly (*p* < .0001) increased in the colitis control with mean level of 165.0 ± 6.45 mU/mg (Figure 6A). Administration of sulphasalazine to the positive control group significantly (*p* < .0001) decreased the activity of MPO to 75.0 ± 2.87 mU/mg compared to the disease control group. PNE also significantly (*p* < .0001) reduced the activity of MPO at all doses with mean values of 130.0 ± 4.08, 97.50 ± 2.50, and 67.50 ± 2.50 mU/mg observed at 30, 100, and 300 mg/kg dose levels, respectively (Figure 6A).

Intrarectal administration of acetic acid resulted in increased lipid peroxidation within the colon. This was observed as significant (*p* < .0001) elevation in levels of MDA in colitis control rats, as opposed to levels in naïve control rats with mean levels 178.2 ± 0.95 M/mg protein and 60.34 ± 0.18 M/mg protein, respectively (Figure 6B). Sulphasalazine significantly (*p* < .0001) reduced the MDA levels to 80.59 ± 0.25 M/mg protein compared to 178.2 ± 0.95 M/mg protein of the colitis control. Likewise, treatment with PNE at all doses reduced MDA levels to 158.1 ± 0.44, 108.4 ± 0.83, and 80.29 ± 0.18 M/mg protein, respectively at 30, 100, and 300 mg/kg dose levels (Figure 6B). Thus, PNE significantly (*p* < .0001) reduced MDA levels compared to the colitis control group.

## 4 DISCUSSION

In this study, the anti‐inflammatory potential of the total crude alkaloidal extract of *P. nitida* seeds was investigated in acetic acid‐induced UC. Following intrarectal administration, acetic acid ionizes in intracellular spaces causing increased intracellular acidification that results in profound destruction of the intestinal epithelial lining.^17^ In addition, acetic acid triggers dysregulated mucosal immune response via ROS‐mediated activation of resident macrophages and neutrophils.^18^ Thus, resulting in a nontransmural inflammation characterized by massive mucosal layer necrosis, hemorrhage, diarrhea, anorexia, malabsorption, progressive weight loss, and intestinal edema.^19^ Experimentally, the acetic acid‐induced UC model is a reproducible model that recreates pathological pathways resembling human UC histologically and biochemically.^20^ As such, screening of medicinal plants using this rodent model may be valuable in identifying potential compounds that may be used effectively in the management of UC.

In this study, the total crude alkaloidal extract of *P. nitida* reduced macroscopic and microscopic disease activity following colonic challenge with acetic acid. This is attributed to PNE's ability to inhibit inflammatory cell infiltration and associated edema, mucosal erythema, erosions, ulceration, diarrhea, and melena. Thus. PNE protects loss of mucosal architecture, mucosal thickening, and depletion of goblet cells. In addition, inhibition of colon edema may imply the ability of PNE to inhibit the production and/or release of inflammatory mediators that alters vascular permeability.^21^ Reduction in vascular permeability and subsequent inhibition of inflammation‐induced decrease in oncotic pressure within blood vessels located in the lamina propria may account for the reduction in colon weight‐to‐length ratio upon treatment with PNE.^21^ Unsurprisingly, Woode et al.^9^ demonstrated that PNE exhibits significant anti‐inflammatory activity in carrageenan‐induced paw edema and adjuvant‐induced arthritis in rats.

In addition to reduction in DAI, treatment with PNE inhibited weight loss following colon injury. Ulcer‐induced related pain stimulates afferent vagal serotonergic nerves and results in anorexia that limits absorption by reducing the passage for chyme through damaged portions of the gastrointestinal tract and may result in malabsorption.^22^ As such, the ability of PNE to reduce mucosal necrosis and associated inflammation and pain may explain its inhibition of weight loss following intrarectal acetic acid administration. Moreover, treatment with PNE may have inhibited the effect of food restriction on iron metabolism, thus an increase hematologic indices such as red cell count and hemoglobin.^23^ In addition, the protection against bowel ulceration following PNE administration may account for the increase in these parameters in addition to platelet levels.^24^

Treatment with PNE reduced oxidative stress and serum expression of pro‐inflammatory mediators. As prime contributing factor to the development of IBD, oxidative and nitrosative stress cause cellular injury which leads to infiltration of the mucosa by inflammatory cells that produce pro‐inflammatory cytokines including TNF‐α and IL‐1β.^25^, ^26^ TNF‐α and IL‐1β are major immune regulatory cytokines that intensify the inflammatory response via activation of a cascade of inflammatory cells, including neutrophils and other white blood cells.^27^ Infiltrated neutrophils produce large amounts of reactive oxygen species which may function as second messenger molecules in activating nuclear factor kappa B (NF‐κB) and p38 MAPK pathways leading to protein dysfunction and apoptosis.^28^ PNE suppressed the production of nitric oxide and increased glutathione levels which may have accounted for the reduction in serum levels of TNF‐α, IL‐1β, and p38 MAPK. Thus, explaining the preservation of mucosal architecture in PNE‐treated groups. This is because, secretion of pro‐inflammatory cytokines such as TNF‐α, IL‐1β, and IL‐6 by macrophages and natural killer cells causes tissue damage with subsequent exposure of the intestinal wall to luminal antigens. This results in strong induction of innate and adaptive immunity which in turn sustain the inflammatory state leading to chronic inflammation and extensive tissue damage.^29^ MAPK, on the other hand is a key regulator of NF‐κB activity which regulates the transcription of genes that encodes several pro‐inflammatory cytokines and nitric oxide.^30^ Interestingly, targeted therapies that reduce the activities of TNF‐α, IL‐1β, and p38 MAPK have been discovered to be effective in patients with IBD.^29^ Thus, the inhibition of colon inflammation by PNE can be attributed, in part, to the suppression of inflammatory mediators via downregulation of p38 MAPK/NF‐κB pathway.

Last, PNE administration suppressed lipid peroxidation following induction of colon injury with acetic acid. It has been hypothesized that, reduced antioxidant activity, such as decreased GSH expression, in active UC is associated with increased colonic lipid peroxidation which can be measured by MPO and MDA levels.^31^ Myeloperoxidase seems to have oxidation potential for chloride ions which results in the creation of a highly cytotoxic neutrophil system that subsequently leads to tissue destruction.^31^, ^32^ As such it can be postulated that PNE protects against mucosal damage by increasing GSH levels which decreases colonic MPO and lipid peroxidation. This is further supported by the reduction in production of MDA, a byproduct of lipid peroxidation, in PNE‐treated colons.

The study, however, has a few limitations. The assessment of markers of inflammation and mitogen activated protein kinase activity using the ELISA kits could have also been done using the colon tissues as well.

## 5 CONCLUSION

The findings from this study demonstrate that PNE improves colon inflammation in acetic acid‐induced colitis by increasing serum glutathione levels while inhibiting nitric oxide production and serum expression of the pro‐inflammatory cytokines, TNF‐α and IL‐1β. PNE suppresses p38 MAPK/NF‐κB pathway and reduces colonic lipid peroxidation by decreasing MDA and MPO levels. Ultimately, PNE improves acetic acid‐induced colitis in rats and shows significant potential as a source of drug lead for use in the treatment of intestinal inflammatory disorders. However, further studies would help establish the local anti‐inflammatory potential of the extract by evaluating the effect of PNE on mediators of inflammation expressed in colonic tissues in colitis.

## AUTHOR CONTRIBUTIONS

**Sarah A. Otu‐Boakye**: Data curation; investigation; writing—original draft. **Kofi O. Yeboah**: Data curation; investigation; methodology; writing—original draft. **Eric Boakye‐Gyasi**: Conceptualization; project administration; supervision; writing—review and editing. **James Oppong‐Kyekyeku**: Conceptualization; project administration; supervision; writing—review and editing. **Prince D. Okyere**: Data curation; investigation. **Newman Osafo**: Conceptualization; formal analysis; funding acquisition; resources; software; supervision; validation; visualization; writing—review and editing.

## CONFLICT OF INTEREST STATEMENT

The authors declare no conflic of interest.

## ETHICS STATEMENT

Ethical clearance for this study was obtained from the Animal Research Ethics Committee (AREC) of KNUST for the work using rodents (reference: KNUST 0014).

## Supporting information

Supporting information.

## Data Availability

The datasets generated during and/or analyzed during the current study are available from the corresponding authors on reasonable request.
